# The risk assessment model of fracture nonunion after intramedullary nailing for subtrochanteric femur fracture

**DOI:** 10.1097/MD.0000000000025274

**Published:** 2021-03-26

**Authors:** ZhengHao Wang, KaiNan Li, ZuChao Gu, HaiQuan Fan, HaiBo Li

**Affiliations:** a Affiliated Hospital of ChengDu University; b Chengdu First people's Hospital; c The Second Affiliated Hospital of Chengdu Medical College; d Shuangliu District First People's Hospital, Chengdu, China.

**Keywords:** calibration, fracture nonunion, logistic regression, nomogram diagram, prediction, subtrochanteric fracture

## Abstract

To investigate the influencing factors of fracture nonunion after intramedullary nailing for subtrochanteric fractures and to construct a risk assessment model.

Based on the multicenter retrospective analysis of 251 patients, all patients were divided into modeling group and verification group. In the modeling group, postoperative fracture nonunion rate, general data, fracture-related factors, surgical reduction-related factors, mechanical and biological factors were calculated, and the influencing factors of fracture nonunion were screened by univariate analysis. Logistic regression model was used for multifactor analysis to construct the risk assessment model. Based on the logistic regression model, the risk prediction model was constructed by drawing the Nomogram diagram. Through the verification group, the influencing factors were evaluated again, and the differentiation and calibration of the model were evaluated. The calibration degree was evaluated by Hosmer-Lemeshow test, goodness of fit test, and calibration curve. The discriminant degree was evaluated by the receiver operating characteristic curve.

Fracture nonunion occurred in 34 of 149 patients in the modeling group. Among the 14 potential influencing factors, univariate analysis and logistic regression analysis showed that postoperative hip varus, intramedullary nail fixation failure, and reduction of fracture with large incision were the risk factors of fracture nonunion. The medial cortex fracture was seen reduced on X-Ray was a protective factor for fracture nonunion, and a regression equation was established. Based on the logistic regression model, the Nomogram diagram is drawn. Twenty-four cases of fracture nonunion occurred in the verification group. The area under the receiver operating characteristic curve was area under curve =0.883 > 0.7, indicating that there was a moderate differentiation to evaluate the occurrence of fracture nonunion after operation. The goodness of fit test: the Hosmers-Lemeshow test (*X*^2^ = 2.921, *P* = .712 > .05) showed that the model had a good calibration.

After intramedullary nailing of subtrochanteric fracture, hip varus, failure of intramedullary nail fixation and wide surgical dissection are the risk factors of fracture nonunion, and the postoperative reduction of medial cortex fracture is protective factor.

National key research and development projects: 2016YFC0105806

## 1 Introduction

Subtrochanteric fractures account for about 25% of all hip fractures, and the age distribution shows a bimodal trend.^[1,2]^ Subtrochanteric fractures are defined as fractures that occur within 5 cm of the lesser trochanter and its distal end.^[3,4]^ Due to difficulty of intraoperative reduction of subtrochanteric fracture, the rate of subtrochanteric fracture nonunion and malunion is high. Studies have shown that the postoperative fracture healing rate of subtrochanteric fracture is about 77% to 99%. The incidence of postoperative complications was as high as 21%.^[5–7]^ Previous studies have suggested that there are many factors affecting fracture nonunion after subtrochanteric surgery, including fracture stability (fracture type).^[1]^ The quality of intraoperative fracture reduction, intraoperative minimally invasive or not, and the bone condition of the patient may also affect the nonunion of the fracture.^[8–11]^At present, there is still a lack of research on the risk factors of fracture nonunion after intramedullary nailing operation, and there is still a lack of fracture nonunion risk assessment model. The risk assessment model is a diagnostic model that predicts the probability of a disease outcome by a combination of risk and protection factors.^[12]^ The model can be used to evaluate risk of disease in clinic. Fracture nonunion after subtrochanteric fracture is often reported, and the revision surgery rate is high. So it is necessary to establish the model for risk assessment to evaluate and predict whether fracture nonunion will occur after intramedullary nail (IMN) operation.

In this project, a multicenter retrospective study was conducted to find out clinical and follow-up data of patients with subtrochanteric fractures treated with intramedullary nailing from February 2014 to January 2018 from a multicenter database containing four hospitals in Chengdu, Sichuan Province. The purpose of this study is to provide a theoretical basis for the prevention of fracture nonunion.

(1) Investigate the risk and protective factors of fracture nonunion after IMN for subtrochanteric fracture;
(2) On basis of factors affecting the fracture nonunion in model group, the regression equation of fracture nonunion is established;
(3) Based on logistic regression model, the Nomogram diagram is drawn, and the calibration and fitting degree of model are evaluated by the verification group.

## 2 Methods

### 2.1 Inclusion and exclusion criteria

The inclusion criteria were as follows:

(1) the patients were treated with IMN for the first time;
(2) the patients were more than 18 years old;
(3) the follow-up time was more than 1 year, and the case and imaging data were complete.

The exclusion criteria were as follows:

(1) fracture caused by primary or metastatic bone tumor;
(2) Patients with fractures around the prosthesis or medial fixation;
(3) intertrochanteric fracture of the femur, which extended to subtrochanteric region of femur.

### 2.2 Details of study design

A multicenter retrospective study was conducted to find out clinical and follow-up data of patients with subtrochanteric fractures treated with intramedullary nailing from February 2014 to January 2018 from a multicenter database containing 4 hospitals in Chengdu, Sichuan Province. All patients were from emergency department and outpatient department. The patients were divided into 2 groups; group 1 (modeling) and group 2 (verification). The modeling group from February 2014 to February 2016 for the construction of risk assessment models, and the verification group from March 2016 to January 2018 for evaluation of effectiveness of the model. Patients in each group were classified according to the occurrence of postoperative fracture nonunion. Patients with complete perioperative and follow-up data should be included as far as possible. Patients lacking follow-up data should inquire about their condition by phone and make an appointment to complete the follow-up imaging data.

### 2.3 The observation and follow-up statistical indicators of all patients are as follows

Subtrochanteric fracture nonunion was defined as persistent pain at the fracture site at 9 months after operation.^[13]^ At the same time, 9 months after operation, the X-ray still showed that there were at least 3 unbridged cortex in the medial, external, anterior, and posterior parts of the fracture.^[14]^

Following are the observation indexes of this study (Table 1):

**Table 1:** The observation indexes of this study.

① Patients’ general condition factors	② Fracture-related factors	③ Surgical reduction related factors	④ Mechanical factors	⑤ Biological factors
1. Age	1. Injury mechanism	1. Unreset displacement distance measured in the anteroposterior position	1. Whether there was hip varus after operation	1. Whether to reduction of fracture with large incision
2. Sex	2. Fracture types (Seinsheimer)	2. Unreset displacement distance measured in the lateral position	2. Whether the medial cortex fracture was seen reduced on X-Ray	2. Whether to use steel wire encircling
3. Osteoporosis	3. Distance between tip of greater trochanter and center of the main fracture line		3. Whether intramedullary nail fixation was invalid	

Description:

①**3:** As described by Sah et al the cortical thickness index was used to assess osteoporosis.^[15]^ The value of lateral X-ray <0.4 cm showed osteoporosis.

②**1:** High or low energy trauma was determined based on the injury mechanism.

②**3:** The distance between the tip of the greater trochanter and the center of the main fracture line was measured on postoperative X-ray.

③**1\2:** The displacement distance of residual fracture after reduction was measured in anteroposterior views and lateral views on X-ray images.

④**1:** An uncorrected hip varus was defined as a varus angle >5°.

**Figure 1:**
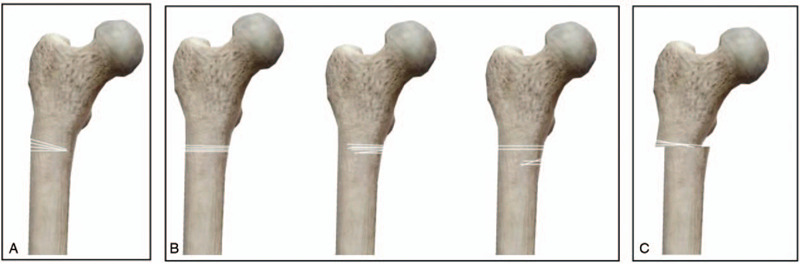
“Medial cortex intact and hip varus” (A), “No medial cortical support without hip varus” (B) and “No medial cortical support and hip varus” (C).

④**2:** Whether the medial cortex fracture was seen reduced on X-Ray (Fig. 1)

④**3:** The failure of IMN fixation was defined as fracture of IMN or loosening of distal locking nail.^[16]^

### 2.4 The participating units are as follows

1. Affiliated Hospital of ChengDu University;
2. Chengdu First people's Hospital;
3. The Second Affiliated Hospital of Chengdu Medical College;
4. Shuangliu District First people's Hospital.

The Affiliated Hospital of ChengDu University ethics committee approved this study (The ethical number:2014-LL-21).

### 2.5 Statistical analysis

#### 2.5.1 Group 1 (modeling)

Univariate analysis was used to screen possible risk factors of fracture nonunion in model group. *T* test was used for measurement data in accordance with normal distribution and homogeneous variance, rank-sum test was used for nonconformity, and chi-square test was used for counting data.

The statistically significant factors described the correlation between fracture nonunion and this factor by Gamma value (γ value). The positive value was positive correlation, the negative value was negative correlation, and the absolute value of γ was close to 1, the greater the correlation was.

The factors with statistical differences were further analyzed by Logistics regression model to construct the risk assessment model. Based on the logistic regression model, the risk prediction model was constructed by drawing the Nomogram diagram by R language software 3.6.1 (Duncan Murdoch, USA).^[28]^

#### 2.5.2 Group 2 (verification)

The calibration and differentiation of model were evaluated by verification group. The calibration degree is evaluated by Hosmer-Lemeshow test, goodness of fit test, and calibration curve. The smaller the value of *X*^2^ is, the larger the *P* value is, and the better the calibration is.^[17]^

The discriminant degree was evaluated by the receiver operating characteristic curve (ROC curve) and the area under curve (AUC). The area value under the curve AUC is between 0.5 and 1.0. The closer AUC is to 1.0, the better prediction of fracture nonunion is. The accuracy of AUC was low at 0.5 to 0.7, accuracy of AUC was moderate at 0.7 to 0.9, and accuracy of AUC was higher at 0.9 to 1.0.^[18]^ The data were analyzed by spss 22.0 (IBM, USA). The inspection level α is 0.05 on both sides.

## 3 Result

### 3.1 Baseline information of the patients

According to the above inclusion and exclusion criteria, a total of 251 patients were included. There were 166 females and 85 males. The age was 20 to 82 years with mean ± SD = 68.95 ± 17.90 years. Seinsheimer classification included type I (n = 10), type II (n = 101), type III (n = 62), type IV (n = 27), and type V (n = 51). According to the healing of subtrochanteric fracture after IMN operation, 251 cases were divided into fracture nonunion group (n = 58) and fracture union group (n = 193). The causes of injury were 63 cases of high energy injury in 251 cases, including 37 cases of traffic injury, 21 cases of high fall injury, 5 cases of heavy object injury. Low energy injury: 188 cases of falls.

#### 3.1.1 Group 1 (modeling)

A total of 149 cases in the modeling group. There were 98 females and 51 males. The age was 20 to 81 years old, with mean ± SD = 68.46 ± 17.22 years old. Seinsheimer classification: type I in 4 cases, type II in 66 cases, type III in 35 cases, type IV in 14 cases, and type V in 30 cases. There were 34 cases in fracture nonunion group and 115 cases in fracture healing group. The causes of injury were high energy injury (n = 35), traffic injury (n = 21), high fall injury (n = 11), heavy object injury (n = 3), and low energy injury (n = 114).

#### 3.1.2 Group 2 (verification)

A total of 102 cases in the verification group. There were 68 females and 34 males. The age was 25 to 82 years old, with mean±SD = 69.68 ± 18.91 years old. Seinsheimer classification: type I in 6 cases, type II in 35 cases, type III in 27 cases, type IV in 13 cases, and type V in 21 cases. According to the fracture healing of trochanter after IMN operation, 102 cases were divided into fracture nonunion group (n = 24) and fracture union group (n = 78). The causes of injury were high energy injury (n = 28), traffic injury (n = 16), high fall injury (n = 10), heavy object injury (n = 2), and low energy injury (n = 74).

### 3.2 Univariate analysis of factors influencing nonunion of fracture

Among the 149 cases in model group, there were 34 cases in fracture nonunion group and 115 cases in fracture healing group. The risk factors were compared between 2 groups.

(1) There was no significant difference in age, sex, and osteoporosis between 2 groups (*P* = .216, .186, .216).
(2) There was no significant difference in the related factors of fracture between 2 groups: injury mechanism, fracture type, distance between the tip of the greater trochanter and the center of the main fracture line (*P* = .364, .054, .192).
(3) The related factors of surgical reduction: the distance of unreset displacement measured in the anteroposterior views, the distance of the unreset displacement measured in the lateral views, the difference was not statistically significant (*P* = .328, .438).
(4) Comparison of mechanical factors between the 2 groups: whether the hip varus was corrected after operation, whether the medial cortex fracture was seen reduced on X-Ray, and whether the distal locking nail was loosened or broken, which led to the failure of IMN fixation, the difference was statistically significant (*P* = .012, .012, .000).
(5) Comparison of biological factors between the 2 groups: Reduction of fracture with large incision, the difference was statistically significant (*P* = .041). There was no statistically significant difference in the use of wire looping (*P* = .564) (Table 2).

**Table 2:** Risk factors of fracture nonunion.

		Fracture nonunion 1	Fracture healing 0	*T* value or *X*^2^ value	*P* value
Age	68.46 ± 17.22	71.68 ± 16.40	67.50 ± 17.42	*t* = -1.243	*P* = .216
Sex					
Male/Female (1/0)	55/94	14/20	41/74	*X*^2^ = 0.344	*P* = .686
Osteoporosis					
Yes/No (1/0)	101/48	20/14	81/34	*X*^2^ = 1.620	*P* = 216
Injury mechanism					
High/ Low energy (1/0)	35/114	10/24	25/90	*X*^2^ = 0.860	*P* = .364
Fracture type (Seinsheimer typing)					
Type I/II/III/IV/V (1/2/3/4)	4/66/35/14/30	0/10/14/4/6	4/56/21/10/24	*X*^2^ = 8.680	*P* = .054
Distance between tip of greater trochanter and center of the main fracture line on X-ray (mm)	90.76 ± 8.99	92.53 ± 7.03	90.23 ± 9.46	*t* = -1.309	*P* = .192
Unreset displacement distance measured in anteroposterior position on X-ray (mm)	4.40 ± 2.65	4.79 ± 3.12	4.29 ± 2.50	*t* = -0.981	*P* = .328
Unreset displacement distance measured in lateral positionon X-ray (mm)	4.13 ± 2.59	4.47 ± 3.13	4.07 ± 2.48	*t* = -0.778	*P* = .438
Postoperative hip varus					
Yes/No (1/0)	47/102	17/17	30/85	*X*^2^ = 6.949	*P* = .012
Reduction of cortex fracture					
Yes/No (1/0)	77/72	11/23	66/49	*X*^2^ = 6.588	*P* = .012
Intramedullary nail fixation is invalid					
Yes/No (1/0)	18/131	16/18	2/113	*X*^2^ = 50.745	*P* < .001
Reduction of fracture with large incision					
Yes/No (1/0)	95/54	27/7	68/47	*X*^2^ = 4.671	*P* = .041
Encircling thread					
Yes/No (1/0)	72/77	18/16	54/61	*X*^2^ = 0.376	*P* = .564

Statistically significant index: the correction of postoperative hip varus was positively correlated with fracture nonunion (γ = 0.478, *P* < .020). There was a negative correlation between the reduction of medial wall and fracture nonunion (γ = -0.476, *P* < .012). The failure of IMN fixation was positively correlated with fracture nonunion (γ = 0.961, *P* < .007). Reduction of fracture with large incision was positively correlated with the nonunion of fracture (γ = 0.454, *P* < .029). The correlation between above 4 indexes and fracture nonunion was statistically significant.

### 3.3 Multivariate logistic regression analysis of risk factors of fracture nonunion

Univariate analysis showed that risk factors of fracture nonunion, such as failure of IMN fixation, postoperative hip varus, reduction of medial cortex fracture, and reduction of fracture with large incision, were analyzed by binary Logistic regression model. These 4 factors were all independent risk factors for fracture nonunion after intramedullary nailing for subtrochanteric fractures. Failure of IMN fixation, postoperative hip varus, and reduction of fracture with large incision were risk factors (β > 0). The Odds Ratio values were 34.446 (*P* **=** .001), 3.241 (*P* **=** . 041), and 3.274 (*P* **=** . 039). Respectively, the medial cortex was seen reduced on X-Ray was protective factor of fracture nonunion (β > 0), and the Odds Ratio value was 0.277. The results were statistically significant (*P* **=** . 021) (Table 3).

**Table 3:** Multivariate analysis of risk factors for fracture nonunion in modeling group.

						95% (CI) confidence interval	
Risk factors	Regression coefficient β value	Standard error S.E value	Wals	*P* value	OR value	Lower limit	upper limit
Failure of intramedullary nail fixation	3.539	0.833	18.055	.000	34.446	6.732	176.262
Postoperative hip varus	1.176	0.576	4.170	.041	3.241	1.048	10.020
Reduction of medial cortex fracture	−1.284	0.554	5.364	.021	0.277	0.093	0.821
Reduction of fracture with large incision	1.186	0.574	4.270	.039	3.274	1.063	10.085
Constant	−2.396	0.550	19.003	.000	0.091	–	

Through logistic regression analysis, the regression equation was established as follows: logit *P* = -2.396 + 3.539 × failure of IMN fixation + 1.176 × postoperative hip varus - 1.284 × reduction of medial wall + 1.186 × reduction of fracture with large incision. (Failure of IMN fixation is = 1, No = 0; Postoperative hip varus is =1, No = 0; Reduction of medial cortex fracture is =1, No = 0; The reduction of fracture with large incision was =1, No = 0), and the probability of nonunion after intramedullary nailing was *P* = exp (logit *P*) / [1 + exp (logit *P*)].

### 3.4 Nomogram diagram based on logistic regression model

**Figure 2:**
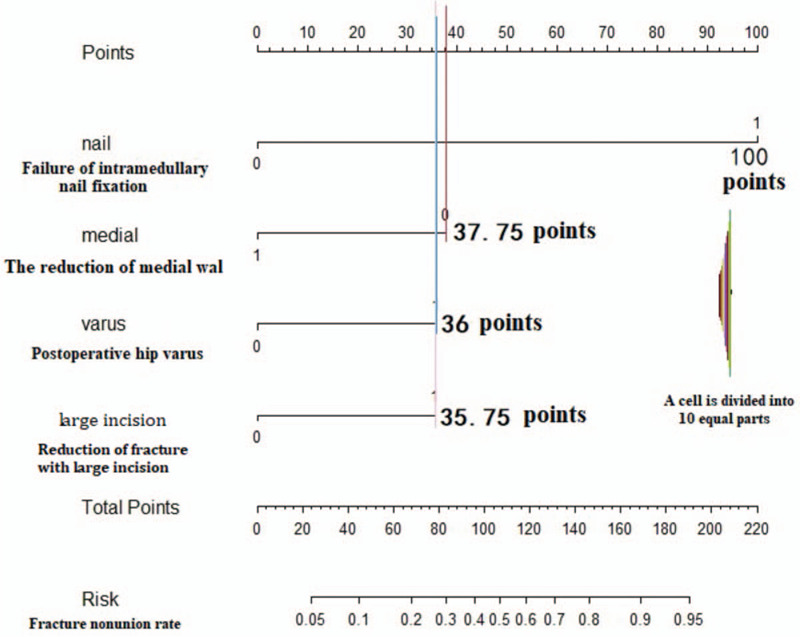
Nomogram diagram of fracture nonunion prediction model.

The R language software was used to assign research indexes to patients with fracture nonunion, and the middle and strong predictive factors (IMN fixation failure) were assigned to 100 points. Then, using regression coefficient values corresponding to 4 statistically significant indexes in logistic regression analysis, the corresponding scores of each index were obtained by R language software program, and the Nomogram diagram for predicting fracture nonunion after intramedullary nailing fixation of subtrochanteric fracture was drawn. In the Nomogram diagram, the corresponding score of each index was summed up, and corresponding fracture nonunion rate was found by total score (Fig. 2).

### 3.5 Verification of risk assessment model for fracture nonunion

**Figure 3:**
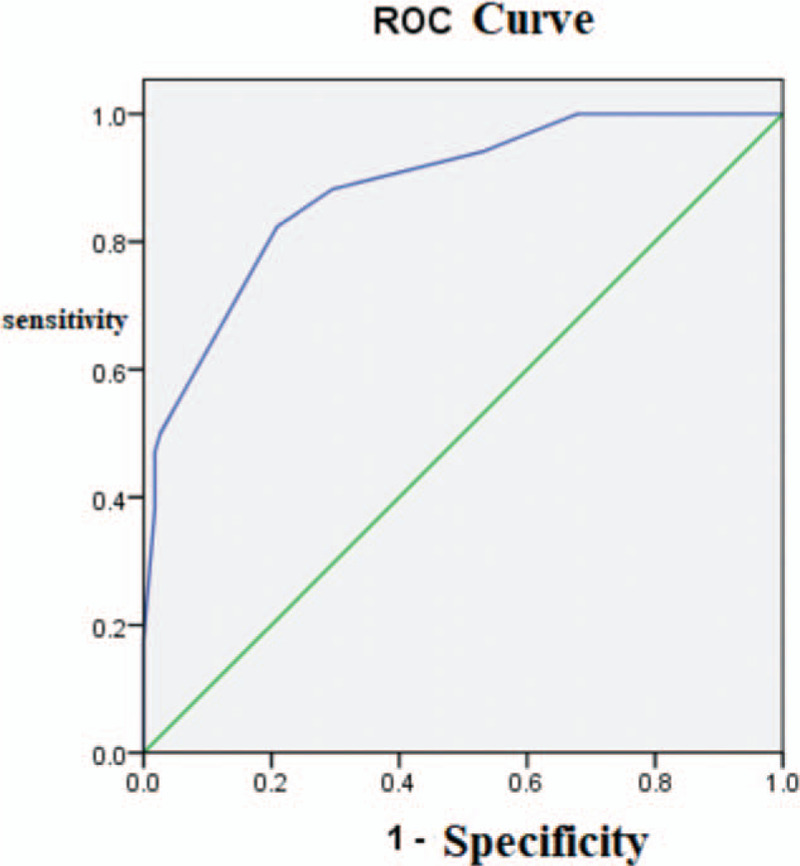
Receiver operating characteristic curve of verification group.

Among the 102 cases in the verification group, 24 cases were nonunion (23.5%). In the modeling group, there were 4 factors affecting fracture nonunion: postoperative hip varus (*X*^2^ = 6.824, *P* = .013), and the medial cortex fracture was seen reduced on X-Ray (X^2^ = 5.976, *P* = .019), the failure of IMN fixation (*X*^2^ = 30.898, *P* < .001), and whether reduction of fracture with large incision (*X*^2^ = 4.895, *P* = .033) (Table 4). It was also proved by statistical analysis in the verification group that it also affected the fracture nonunion after subtrochanteric fracture IMN fixation. The results were statistically significant. In the verification group, the discrimination and fitting degree of the prediction efficiency of the binary logistic regression model were analyzed. The differentiation analysis showed that the classification evaluation model predicted the ROC curve of fracture nonunion after subtrochanteric fracture IMN (Fig. 3). The ROC curve takes the specificity as transverse coordinate (false positive rate) and the sensitivity as longitudinal coordinate (true positive rate). The area under ROC curve AUC was 0.883 > 0.7, <0.9, and *P* < .001, which was statistically significant. The risk assessment model has a moderate degree of differentiation in predicting the occurrence of fracture nonunion. The goodness of fit test (Hosmer-Lemeshow) showed that the model had a good calibration degree *X*^2^ = 2.921, *P* = .712 > .5. the results showed that the model had a good calibration degree.

**Table 4:** Univariate analysis of risk factors for fracture nonunion in the validation group.

102 cases of verification group		Fracture nonunion1	Fracture healing 0	*X*^2^ value	*P* value
Postoperative hip varus					
Yes/No (1/0)	33/69	13/11	20/58	*X*^2^ = 6.824	*P* = .013
Reduction of medial cortex fracture					
Yes/No (1/0)	52/50	7/17	45/33	*X*^2^ = 5.976	*P* = .019
Intramedullary nail fixation is invalid					
Yes/No (1/0)	13/89	11/13	2/76	*X*^2^ = 30.898	*P* < .001
Reduction of fracture with large incision					
Yes/No (1/0)	61/41	19/5	42/36	*X*^2^ = 4.895	*P* = .033

## 4 Discussion

### 4.1 Main findings of the present study

**Figure 4:**
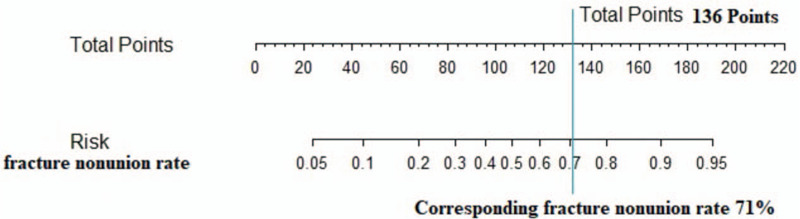
A case of fracture nonunion rate corresponding to total score of Nomogram diagram.

The purpose of this study was to explore the relationship between postoperative fracture nonunion and related factors, including patient-related, fracture-related, surgical reduction-related, mechanical-related, and biological related factors. Among the above 14 factors, one biological related factor: reduction of fracture with large incision during operation. Three mechanical factors: incomplete reduction of medial cortex fracture, uncorrected hip varus deformity, and failure of IMN fixation were related to postoperative fracture nonunion. The logistics regression model was established, on the basis of which the Nomogram diagram was drawn to evaluate the nonunion rate of subtrochanteric fracture: 100 points for IMN failure, 37.75 points for the medial cortex fracture is not seen reduced on X-Ray, 36 points for hip varus deformity after operation. The score of reduction of fracture with large incision was 35.75 points. Example: in 1 patient with subtrochanteric fracture after intramedullary nailing, the hip varus deformity was not corrected (36 points), IMN fixation failure (100 points), in reduction of fracture with large incision (0 points), the medial cortex was seen reduced on X-Ray (0 points), total score of 136 points. The corresponding fracture nonunion rate was 71% **(**Fig. 4**)**.

### 4.2 Implication and explanation of findings

#### 4.2.1 The medial cortex fracture is seen reduced on X-Ray can reduce the incidence of fracture nonunion

IMN fixation for shaft and metaphyseal fractures is one of the most successful techniques in orthopedics.^[19]^ It promotes fracture healing by providing relative stability, while limited incision maintains the blood supply, especially the periosteal blood supply.^[20]^ However, studies have shown that the fracture ununion rate of subtrochanteric fracture is relatively high after IMN fixation.^[9]^ The discovery can be explained by different biological and mechanical characteristics of the subtrochanteric region. The medial subtrochanteric cortex bears bending forces up to 8.2 MPA under physiological load.^[11,21]^ If the medial cortex is not seen reduced on X-Ray during the operation, the stress will be completely borne by the IMN until the fracture heals. Lack of medial cortical support is a risk factor for fracture nonunion after subtrochanteric fracture.^[13]^ Anatomical reduction will restore medial cortical support against bending force and varus torque.

In this study, the medial cortex fracture was seen reduced on X-Ray after intramedullary nailing was negatively correlated with fracture nonunion (γ coefficient = -0.476), which was the protective factor of fracture nonunion. The fracture nonunion rate of unreduced medial wall was 38.33% (23/60). The defect of intramedullary fixation in the treatment of subtrochanteric fracture is difficult for reduction of medial wall. For the subtrochanteric fracture of femur, the stability of medial cortex should be restored as far as possible during intramedullary fixation, which will help to reduce the occurrence of fracture nonunion. However, this study shows that in order to pursue anatomical reduction and reduction of fracture with large incision, destroying the periosteal blood supply will increase the probability of fracture nonunion, so steel wire or cable can be reinforced with as little trauma as possible on basis of IMN.

#### 4.2.2 Postoperative hip varus deformity may provide an incidence of nonunion of fractures

Previous studies have shown that hip varus deformity is a risk factor for nonunion of subtrochanteric fracture.^[22]^ The decrease of cervical trunk angle in hip varus deformity leads to the increase of bending stress in medial subtrochanter cortex under same physiological load. This stress also needs to be borne by IMNs until the bone heals. The subtrochanteric region is smaller than the intertrochanteric medullary cavity, the fracture area accounts for a higher proportion, and the probability of hip varus deformity is relatively increased.^[9]^ Hip varus deformity was more correlated with subtrochanteric Seinsheimer type V fracture.^[23]^ During the operation, according to anatomical structure of each patient, it is very important to select the correct entry point.

According to our clinical experience and combined with the literature, we suggest the following two aspects to avoid hip varus. First of all, IMN should not be used as a reduction tool for subtrochanteric fracture. It should be corrected by traction bed traction and manual reduction before insertion of IMN, and fracture reduction should be achieved as far as possible.^[24]^ After the traction reduction was determined by intraoperative C-arm fluoroscopy, the correct nail entry point was identified. Secondly, for the proximal curved IMN, the ideal needle entry point can not be on the outside of the apex of the greater rotor. In order to avoid varus deformity, it is recommended that the entry point be close to inside of the apex of greater trochanter, and can choose the intersection point of piriform fossa and long axis of femur as the entry point.

In this study, hip varus deformity after intramedullary nailing was positively correlated with fracture nonunion (γ = 0.478). The ununion rate of postoperative varus deformity was 36.96% (17/46). Displacement of fracture fragments caused by adduction, abduction, external rotation, and flexor muscle activity around the hip. During the intraoperative reduction of the proximal femoral fracture fragments, they were pulled by the gluteus medius and small muscles, and were pulled by the iliopsoas muscle during flexion and external rotation. On the other hand, adductor muscle pull fracture fragments inward, increase fracture displacement, intraoperative reduction is more difficult, especially the reduction of medial wall is difficult, hip varus probability is increased, which is the main cause of intraoperative and postoperative complications. The reduction of fracture is the focus of treatment.

#### 4.2.3 Reduction of fracture with large incision may provide an incidence of nonunion of fractures

A number of studies have shown that blood flow rate in subtrochanteric region is low,^[13,16]^ and bone segment with poor blood supply distribution is highly correlated with fracture nonunion rate. The conclusion of this study confirms this view. The reduction of fracture with large incision is positively correlated with the nonunion of fracture. The nonunion rate of fracture was 28.13% (27/96). There is growing evidence that minimizing the destruction of periosteal blood supply is as important as restoring medial cortical support and preventing hip varus.^[11,21]^ The periosteal blood supply in subtrochanter region is circular.^[25]^ The blood supply in the trochanter region can be preserved by steel wire encircling.^[22]^ The application of circumferential steel wire in rotor region is safe and valuable, and is beneficial to the reduction and stability of fracture. Reduction of fracture with large incision destroys not only the periosteal blood supply, but also the organization of the hematoma and osteotylus, as well as the surrounding soft tissue and ligaments, further affecting stability of subtrochanter. The advantage of IMN lies in minimally invasive reset, and to protect the periosteal blood supply.^[13]^ This study showed that reduction of fracture with large incision was positively correlated with fracture nonunion (γ = 0.454).

#### 4.2.4 IMN fixation may provide an incidence of nonunion of fractures

In this study, the failure of IMN is positively correlated with fracture nonunion, and the correlation coefficient is γ = 0.961. The fractures nonhealing with failure of IMN fixation was 88.89% (16/18). One of main findings of our study was that three of 4 risk factors for subtrochanteric fracture nonunion were associated with mechanical factors, including increased load of IMN (hip varus deformity). The stability of subtrochanteric fracture decreased (lack of medial cortical support) or the stability of the whole mechanical structure decreased (IMN fixation failure). The blood supply of bone and soft tissue was seriously damaged (reduction of fracture with large incision), and fracture non-healing rate increased with risk factors. The failure of IMN fixation is a strong predictor of fracture nonunion.

The failure of IMN fixation includes fracture or loosening of IMN and distal locking nail in first 12 weeks after operation. Studies have shown that in treatment of intertrochanteric fracture with IMN, the fixing rod (screw blade) passes through intertrochanteric fracture, and most of stress concentrated in fracture area is borne by the main nail and the fixing rod. It also provides three-point fixation for fracture area, which is relatively stable. However, the fixation rod of IMN for subtrochanteric fracture is located at proximal of fracture, and the stress at fracture is concentrated in main nail. The stress in fracture area is too concentrated and there is no three-point fixation. The probability of IMN breakage increased.^[26]^

Johnson et al^[27]^ analyzed the risk factors for fracture failure of proximal femoral nail fixation within 10 years by logistic regression, it was concluded that young patients with low ASA score of American Anesthesiologist Association had the highest risk of nail breakage. Subtrochanteric fracture and pathological fracture are independent risk factors for fracture failure of IMN. For patients with subtrochanteric fracture, ASA score should be given before operation, and the possibility of pathological fracture caused by cancer metastasis and osteoporosis should be excluded. For patients with subtrochanteric fracture after intramedullary nailing, patients with the above risk factors should be closely followed up, timely review X-ray, avoid falling again, avoid violent activity, until fracture healing. The elderly patients with osteoporosis diagnosed before operation should be treated with regular anti-osteoporosis treatment.

### 4.3 The strengths and limitations in this study

The strengths of this study lies in the multi-center study and the relatively large sample size, so the error of experimental results is relatively reduced. In this study, the nonunion rate of subtrochanteric fracture after intramedullary nailing was directly predicted by regression formula and nomo chart risk factor score. Through this risk model, the incidence of fracture nonunion was predicted to some extent. There are still many limitations in this study:

(1) This study is a retrospective study, so the role of 4 risk factors in predicting fracture nonunion is still insufficient.
(2) Although the cases of subtrochanteric fracture included in this study are multicenter studies, all of these cases come from the same area, and there may be deviation in the establishment of risk prediction model. Future research can increase the sample size or include multicenter data from all over the country to reduce the deviation.
(3) The risk assessment model did not assess the potential risk factors of patients themselves, such as smoking, diabetes and drug intake.
(4) The average age of the patients was relatively high (Range 20–82 years old with mean ± SD 68.95 ± 17.90 years). The main mechanism of injury was low energy trauma, and the rate of osteoporosis was high. Therefore, the results of this study are weak in predicting high-energy subtrochanteric fracture nonunion in young patients and need to be further studied.

## 5 Conclusion, recommendation, and future directions

Nonunion femoral subtrochanteric fracture is affected wide surgical dissection, postoperative coxa vara and mechanical failure of the nail, while reduction and reconstruction of the medial cortex fracture is a favorite factor for healing. The risk assessment model has moderate differentiation and good calibration, which can provide reference for the risk assessment of fracture nonunion. Future studies should focus on the prospective study of this model to verify the accuracy of the fracture nonunion model.

## Author contributions

**Conceptualization:** ZhengHao Wang, Haiquan Fan, Haibo Li.

**Data curation:** ZuChao Gu, Haiquan Fan, Haibo Li.

**Formal analysis:** ZuChao Gu, Haiquan Fan, Haibo Li.

**Writing – original draft:** KaiNan Li.

**Writing – review & editing:** KaiNan Li.
